# Acoustic Source Tracking Based on Probabilistic Data Association and Distributed Cubature Kalman Filtering in Acoustic Sensor Networks

**DOI:** 10.3390/s22197160

**Published:** 2022-09-21

**Authors:** Yang Chen, Yideng Cao, Rui Wang

**Affiliations:** School of Microelectronics and Control Engineering, Changzhou University, Changzhou 213164, China

**Keywords:** acoustic source tracking, distributed acoustic sensor networks, distributed cubature Kalman filter, probabilistic data association

## Abstract

A probabilistic data association-based distributed cubature Kalman filter (PDA-DCKF) method is proposed in this paper, whose performance on tracking single moving sound sources in the distributed acoustic sensor network was verified. In this method, the PDA algorithm is first used to sift the observations from neighboring nodes. Then, the sifted observations are fused to update the state vectors in the CKF. Since nodes in a sensor network have different reliabilities, the final tracking result integrates the estimations from the local nodes, which are weighted with the parameters depending on the mean square error of the estimation and the energy of the received signal. The experimental results illustrated that the proposed PDA-DCKF method is superior to the other DCKF methods in tracking sound sources even under severe noise and reverberant conditions.

## 1. Introduction

The problem of acoustic source localization and tracking has always been one of the research hotspots in the field of speech processing. It has been widely used in many aspects, such as audio and video conferencing systems, human-computer interaction and speech enhancement, etc. [1,2,3,4]. Traditional acoustic localization and tracking methods usually require the microphone array to have a regular geometric structure, and generally use a centralized data processing method [5]. With the continuous advancement of technology, some traditional microphone arrays gradually show some deficiencies. The distributed microphone network has attracted more and more research work because it has no strict restrictions on the arrangement of microphones, and is a network composed of multiple nodes arbitrarily distributed in space, usually each node contains a set of microphones [6,7,8,9,10].

So far, there have been many studies on acoustic source localization using distributed microphone networks [11]. But they only locate the acoustic source based on the current observations of multiple microphones, which can locate the acoustic source when the background noise and reverberation are small. In noisy and reverberant environments, spurious observations may even mask observations from real acoustic sources, degrading localization performance. To avoid this problem, a Bayesian filter [12] combined the current observation with a series of past observations for current position estimation, which is more effective for dealing with the adverse effects of noise or reverberation. Theoretically, Bayesian filters describe the tracking problem with a state-space model that includes a dynamic model that describes the motion of the target and an observation model that describes the relationship between the observations and the state of the acoustic source. When the state space model is linear and Gaussian, Kalman filter can replace Bayesian filter. However, in acoustic source tracking scenarios, the observation function is usually nonlinear, and some conditions and properties applicable to linear systems no longer hold, and the performance of the Kalman filter may be severely degraded.

Extended Kalman filter (EKF) was first proposed in [13] and is the simplest and most widely used nonlinear filtering method. EKF only intercepts the first-order term in the linearization process to approximate the system function, but its error is relatively large. In order to solve this problem, the literature [14] proposed iterative extended Kalman filter (IEKF), which can improve the accuracy of EKF through several iterations. However, when the nonlinearity of the system model is very strong, whether it is EKF or IEKF, their effect is not good, and there are disadvantages such as poor stability and easy divergence. The particle filter (PF) is a Monte Carlo implementation of a Bayesian filter that approximates the state by a series of weighted particles extracted from the proposal function [15]. PF can handle nonlinear and non-Gaussian situations well, and many PF-based sound source tracking methods have been developed. Vermaak and Blake [16] first introduced PF to sound source tracking. The particle filter method breaks through the limitations of linearity and Gaussian, but its computational load is too large. In practical applications, particle filtering will only be considered when the approximate filter fails. The Sigama point Kalman filter method [17] was similar to the idea of particle filter. It does not use the method of approximating nonlinear systems, but directly uses the real system and observation model by selecting a set of effective deterministic sampling sets, namely Sigama points, which can achieve second-order accuracy. According to the different selection methods of Sigama points, it can be divided into UKF, CKF, QKF and so on. In general, several Gaussian filtering methods introduced above are centralized, that is, the data of all nodes is collected and transmitted to the central processing unit to perform the task of acoustic source tracking. This method is generally unreliable, as any failure of the central processor renders the entire network untraceable.

In order to solve the unreliable problem of centralized methods, many distributed methods have been developed for sound source tracking. No central processor is needed in the distributed method, and all nodes realize the estimation of the global state only by exchanging data with their neighbors. In reference [18], a distributed extended Kalman particle filter (DEKPF) for speaker tracking was developed, which combined the current TDOA observations into EKF to propose particle filter. In reference [19], a distributed particle filter (DPF) was proposed, which applied the improved iterative covariance intersection (MICI) algorithm and interactive multiple model (IMM) to speaker tracking in distributed microphone networks. In reference [20], a distributed iterative EKF was proposed to estimate the time-varying speaker position in the microphone array. In reference [21], a Distributed Unscented Kalman Filter (DUKF) is proposed to overcome the nonlinearity of the measurement model in speaker tracking. The time difference of arrival (TDOA) was used as the observation and then the distributed IMM-UKF was used to track the location of the sound source.

In the actual environment, the existence of noise or reverberation usually produces unreliable observations with false peaks, which may lead to serious performance degradation. Usually, the current observations contained in these methods are only extracted from the largest peak value of a certain observation function. In some bad cases, the peak value related to the real acoustic source may be masked by the stray acoustic source. Therefore, it is more reasonable to extract multiple observations from the observation function, rather than one observation, and then incorporate it into the above tracking scheme. Probabilistic Data Association (PDA) [22] is an effective method to combine multiple observations into Kalman filter state update, which has been proved to be suitable for target tracking in clutter environment. In reference [23], an improved distributed unscented Kalman particle filter (DUKPF) was proposed to track a single moving acoustic source using a distributed microphone network in noise and reverberation environments. This method proposed to extract multiple observations from the observation function of each node and combined them into the status update of UKF through probabilistic data association (PDA) technology, so as to generate PDA-UKF, and then brought in particle filter. In reference [24], a microphone array network distributed multi speaker tracking method based on tasteless particle filter and data association was proposed. The available observations were associated with each speaker at each node using data association technology to track the speaker. Reference [25] proposed a volume information filter based on joint probabilistic data association (JPDA) for multi acoustic source tracking based on distributed acoustic vector sensor (AVS) array, in which JPDA was used to deal with the correlation between observations and targets. Issues related to multi-source tracking are beyond the scope of this article. However, most of particle filter-based methods require excessive computational costs, which limits them in real-time applications. Besides, in existing speaker tracking methods, the PDA algorithm is applied to sift the observations without considering the information from neighboring nodes.

Probabilistic data association with cubature Kalman filtering are combined in this paper, and they are applied to the problem of single-acoustic source tracking in noisy and reverberant environments with distributed acoustic sensor networks. The contributions of this paper are as follows:

- Combining the cubature Kalman filter (CKF) with PDA, the probabilistic data association-cubature Kalman filter (PDA-CKF) was developed. In PDA-CKF, multiple possible observations were merged into the state update of CKF by the PDA technique.
- In this paper, PDA-CKF was applied to the distributed acoustic sensor network, and the probabilistic data association-distributed cubature Kalman filter (PDA-DCKF) was developed by combining the observation information of each node’s neighbor nodes in the network.
- Considering the reliability of the local state, it was proposed to combine the mean square error (MSE) of the position estimation of each node and the received signal energy to adjust the weighting coefficient of distributed acoustic sensor data fusion. In this way, the local state of high-quality nodes is enhanced, and each node can achieve global consistency and good speaker tracking performance.

The structure of this paper is as follows. Section 2 presents the problem formulation, background knowledge, and some prior knowledge of acoustic source tracking. Section 3 first introduces the single-node PDA-CKF and then details the distributed PDA-DCKF. Section 4 presents the experimental results and discussion. Section 5 summarizes some conclusions.

## 2. Background Knowledge

### 2.1. Problem Formulation

**Figure 1:**
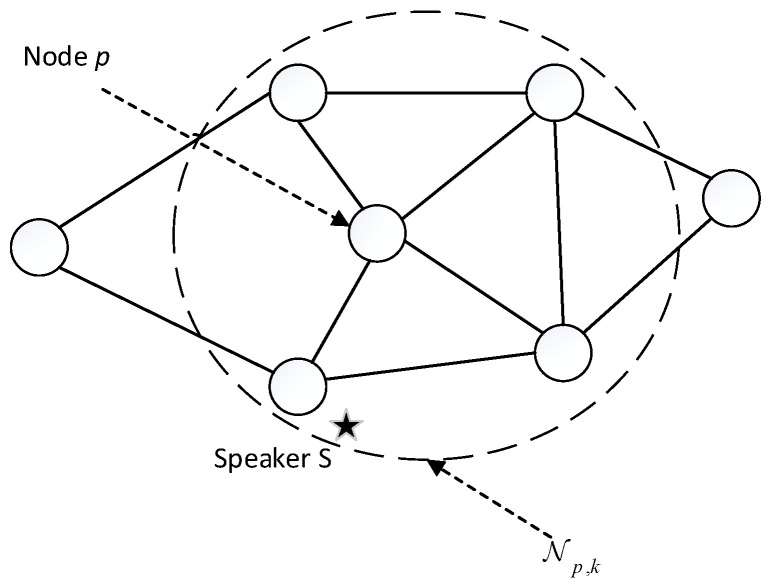
Diagram of speaker tracking in the distributed acoustic sensor network.

Consider a distributed sensor network with N nodes deployed as shown in Figure 1. The positions of the nodes can be obtained in advance by calibration [26]. Each node in the DMA consists of two microphones at distance L. All nodes of the network are modeled as vertices of the graph G1=(ε,υ), where υ={1,2,…,N} is the vertex set, ε⊂{(p,q)|p,q∈υ} is the edge set, and (p,q)∈ε represents the network’s communication constraints, i.e., node p can send information to node q, and vice versa. Let $N_{p,k}=\{q\in \upsilon |(p,q)\in \varepsilon \}\cup \{p\}$ denote the set of neighbors of node p at time k, where a node is a neighbor of itself certainly.

### 2.2. Signal Model and TDOA Estimation

In acoustic sensor networks, the discrete-time signal acquired by the l−th microphone (l=1,2) of node p can be modeled as [23]

(1) $$y_{p,l}(t)=h_{p,l}(t)*y(t)+e_{p,l}(t),\forall p\in \upsilon$$

where t is the discrete-time index, $h_{p,l}(t)$ is the room impulse response (RIR) between the microphone and the acoustic source, ∗ denotes the convolution operator, y(t) is the source signal, and $e_{p,l}(t)$ is the additive noise.

Traditionally, the generalized cross-correlation function (GCC) [27] is used for TDOA estimation. Assuming that $Y_{1}(k)$ and $Y_{2}(k)$ are the acoustic signal received by a microphone pair at time k and $Y_{l}(f)=FFT\{Y_{l}(k)\},l=1,2$ is the frequency domain representation of the corresponding acoustic signal in a time frame, the generalized cross-correlation function of the acoustic signal received by the microphone pair is

(2) $$R_{12}(\tau )=\int_{-\infty }^{+\infty }\frac{Y_{1}(f)Y_{2}^{*}(f)}{\left|Y_{1}(f)Y_{2}^{*}(f)\right|}e^{j2\pi f\tau }df$$

where $Y_{1}(f)$ and $Y_{2}(f)$ represent the frequency-domain microphone signals at the node, and ∗ represents the complex conjugation operation. Therefore, the delay estimation is [27]

(3) $$\hat{\tau }=\int_{-\infty }^{+\infty }\underset{\tau \in [-\tau \max,\tau \max]}{\arg\max}R_{12}(\tau )$$

τmax is the largest time delay estimation.

However, in the real indoor environment, reverberation and noise will bring false maxima of $R_{12}(\tau )$ and obtain invalid TDOA estimation. In order to solve this problem, the local largest of the first Q largest peaks of $R_{12}(\tau )$ are taken as the candidate measurement value of multiple TDOA of node p at time k. In this paper, multiple TDOA observations were extracted through a two-step selection process, taking node p as an example [23].

(1) Select Q delays according to the peak amplitude of the GCC, i.e.,

(4) $$z_{p,k}=[{\tilde{\tau }}_{p,k}^{\left(1\right)},{\tilde{\tau }}_{p,k}^{\left(2\right)},\ldots ,{\tilde{\tau }}_{p,k}^{\left(Q\right)}]$$

where ${\tilde{\tau }}_{p,k}^{\left(i\right)}$ is the delay of node p related to the i−th largest peak of $R_{12}(\tau )$ at time k.

(2) Further, select $m_{p,k}$ observations from (4) as local observations, and the selection rules are shown in Section 3.

(5) $${\overset{⌣}{z}}_{p,k}=[{\tilde{\tau }}_{p,k}^{\left(1\right)},{\tilde{\tau }}_{p,k}^{\left(2\right)},\ldots ,{\tilde{\tau }}_{p,k}^{\left(m_{p,k}\right)}]$$

where each delay ${\hat{\tau }}_{p,k}^{\left(j\right)},j=1,2,\ldots ,m_{p,k}$ is deemed as a TDOA candidate.

### 2.3. Dynamic Model of Acoustic Source

Without loss of generality, the two-dimensional tracking is considered herein, since the height of a moving acoustic source would usually not change significantly. Speakers move in a room with a distributed acoustic sensor network, and Langevin model [24] can accurately and simply describe the time-varying position of speakers. At time k, the state of the speaker is defined as $x_{k}={\left[x_{k},y_{k},{\dot{x}}_{k},{\dot{y}}_{k}\right]}^{T}$, where ${\left(x_{k},y_{k}\right)}^{T}$ and ${\left({\dot{x}}_{k},{\dot{y}}_{k}\right)}^{T}$ represent the position and moving speed of the speaker, respectively. In this model, the speaker’s motion in the Cartesian coordinate system is considered to be independent and modeled as [23]

(6) $$x_{k}=\left[\begin{array}{cc} I_{2} & a\Delta T⊗I_{2}\\ 0 & a⊗I_{2} \end{array}\right]x_{k-1}+\left[\begin{array}{cc} b\Delta T⊗I_{2} & 0\\ 0 & b⊗I_{2} \end{array}\right]u_{k-1}$$

where $a=e^{-\beta \Delta T}$, and $b=\bar{\upsilon }\sqrt{1-a^{2}}$; β and $\bar{\upsilon }$ are the rate constant and the steady velocity parameter, respectively. $I_{s}$ denotes the s-order identical matrix, ⊗ stands for the Kronecker product, ΔT is the sampling period for position estimation, and $u_{k-1}$ is the zero-mean white Gaussian noise with identity covariance matrix, which describes the uncertainty of the acoustic source motion.

### 2.4. Bayesian Framework for Speaker Tracking

Bayesian filtering is the basis of Kalman filtering. This section briefly reviews the basic principles of the Bayesian filtering algorithm.

Assuming that the state variable at time k is $x_{k}\in {\mathbb{R}}^{p}$ and its observation value is $y_{k}\in {\mathbb{R}}^{q}$, where ${\mathbb{R}}^{n}$ represents the n-dimensional real vector space, the state equation and observation equation are expressed as [21]:

(7) $$x_{k+1}=f_{k}(x_{k})+\Gamma_{k}w_{k}$$

(8) $$y_{k}=h_{k}(x_{k})+v_{k}$$

where $f_{k}(\cdot )$ is the nonlinear state transfer function, $h_{k}(\cdot )$ is the nonlinear observation function, $\Gamma_{k}$ is the noise transfer matrix, $w_{k}$ is the process noise, and $v_{k}$ is the observation noise, which meets [21]

(9) $$E\left\{\left[\begin{array}{c} w_{k}\\ v_{k} \end{array}\right]{\left[\begin{array}{c} w_{l}\\ v_{l} \end{array}\right]}^{T}\right\}=\left[\begin{array}{cc} Q_{k}\delta_{k,l} & 0\\ 0 & R_{k}\delta_{k,l} \end{array}\right]$$

where the superscript T represents the transpose of the matrix, E{⋅} represents the expected operator, and $\delta_{k,l}$ represents the Kronecker delta function. $Q_{k}$ and $R_{k}$ are the covariance matrices of noise $w_{k}$ and $v_{k}$, respectively, and it is assumed that they are both positive definite.

The Bayesian filtering problem is to infer the estimated value of the state variable $x_{k}$ at time k given the observation information $y_{1:k}=\{y_{1},\ldots ,y_{k}\}$ at time k, i.e., to estimate the posterior probability density $p(x_{k}|y_{1:k})$. Assuming that the initial probability density function $p(x_{0})$ of the state variable is known as prior knowledge, the posterior probability density $p(x_{k}|y_{1:k})$ can be obtained recursively by the following equations [20]:

(10) $$p(x_{k}|y_{1:k-1})=\int p(x_{k}|x_{k-1})p(x_{k-1}|y_{1:k-1})dx_{k-1}$$

(11) $$p(x_{k}|y_{1:k})=\frac{p(y_{k}|x_{k})p(x_{k}|y_{1:k-1})}{p(y_{k}|y_{1:k-1})}$$

In Equations (10) and (11), the state transition probability density function $p(x_{k}|x_{k-1})$ is defined by the state equation; the observation likelihood probability density function $p(y_{k}|x_{k})$ is defined by the observation equation.

## 3. Improved Distributed Cubature Kalman Filter

In the CKF, the observation corresponding to the largest peak of the observation function is used for the state update. This approach works well under moderate acoustic environments, while its performance degrades in severe noise and reverberation conditions because the spurious peaks from noise or reverberation may cover up the peaks from real acoustic sources. To alleviate this problem, multiple observations are selected from the multiple local maxima of the observation function. A general framework for state updates that integrates multiple possible observations is provided by the probabilistic data association (PDA). Inspired by this idea, the probabilistic data association-cubature Kalman filter (PDA-CKF) was derived in this paper. Next, PDA-CKF was used for acoustic source tracking in distributed acoustic sensor networks, and an improved PDA-DCKF algorithm was developed. The observations of multiple nodes in the neighborhood are filtered by PDA and then merged into the state update of CKF to integrate the information of multiple nodes to realize distributed tracking.

Before introducing PDA-CKF, the preliminary knowledge of cubature Kalman—cubature point set $\left\{\xi_{i},\omega_{i}\right\}$ [28]—should be introduce first.

The standard Gaussian weighted integral is calculated using the spherical-radial cubature rule, i.e., [28]

(12) $$E[x|z]=\int_{R}f(x)N(x;0,P)dx\approx \sum_{i=1}^{2n}\omega_{i}f(\xi_{i})$$

In Equation (12), f(⋅) is the nonlinear state transfer function or observation function, n is the dimension of the state variable, N(x;0,P) is a Gaussian distribution function with a mean of zero and a variance of P, and $\xi_{i}$ is the cubature points.

(13) $$\xi_{i}=\sqrt{n}{\left[1\right]}_{i},i=1,2,\ldots ,2n$$

(14) $$\omega_{i}=\frac{1}{2n}$$

${\left[1\right]}_{i}$ represents the point set of n (n-dimensional state) dimensional space, i.e.,

(15) $${\left[1\right]}_{i}=\left\{\left(\begin{array}{c} 1\\ 0\\ \vdots \\ 0 \end{array}\right),\left(\begin{array}{c} 0\\ 1\\ \vdots \\ 0 \end{array}\right),\ldots ,\left(\begin{array}{c} 0\\ 0\\ \vdots \\ 1 \end{array}\right),\left(\begin{array}{c} -1\\ 0\\ \vdots \\ 0 \end{array}\right),\left(\begin{array}{c} 0\\ -1\\ \vdots \\ 0 \end{array}\right),\ldots ,\left(\begin{array}{c} 0\\ 0\\ \vdots \\ -1 \end{array}\right)\right\}$$

### 3.1. PDA-CKF Algorithm

(a) Initialization

When k=0, assuming $x_{0}\sim N({\bar{x}}_{0},P_{0})$, the initial value of the process noise and observation noise matrix are set to ***Q***_0_ and ***R***_0_, respectively. Then, the optimal initialization of the filter is

(16) $$\begin{array}{l} {\hat{x}}_{p,0|0}={\bar{x}}_{0}\\ {\hat{P}}_{p,0|0}=P_{0} \end{array}$$

(b) State Prediction

For each node p, the state estimate and covariance matrix ${\hat{x}}_{p,k-1|k-1},{\hat{P}}_{p,k-1|k-1}$ at time k−1 are given, and the positive definite noise matrix $Q_{p,k-1},R_{p,k-1}$ are given. Using Equations (13) and (14), the state predicted cubature points $\chi_{p,k-1|k-1}^{i}$ is calculated as:

(17) $$S_{p,k-1|k-1}=\sqrt{{\hat{P}}_{p,k-1|k-1}}$$

(18) $$\chi_{p,k-1|k-1}^{i}={\hat{x}}_{p,k-1|k-1}+S_{p,k-1|k-1}\xi_{i},i=1,2,\ldots ,2n$$

According to the state transition model, the cubature points are propagated nonlinearly, i.e.,

(19) $$\chi_{p,k|k-1}^{i}=f(\chi_{p,k-1|k-1}^{i}),i=1,2,\ldots ,2n,p=1,2,\ldots ,N$$

where n represents the dimension of the state variable, and N represents the number of nodes in the distributed acoustic sensor network. At this time, the state prediction ${\hat{x}}_{p,k|k-1}$ and its error matrix ${\hat{P}}_{p,k|k-1}$ are calculated as:

(20) $${\hat{x}}_{p,k|k-1}=\frac{1}{2n}\sum_{i=1}^{2n}\chi_{p,k|k-1}^{i},i=1,2,\ldots ,2n,p=1,2,\ldots ,N$$

(21) $${\hat{P}}_{p,k|k-1}=\frac{1}{2n}\sum_{i=1}^{2n}(\chi_{p,k|k-1}^{i}-{\hat{x}}_{p,k|k-1}){\left(\chi_{p,k|k-1}^{i}-{\hat{x}}_{p,k|k-1}\right)}^{T}+Q_{p,k},p=1,2,\ldots ,N$$

(c) Status Update

From the estimated ${\hat{x}}_{p,k|k-1}$ and variance ${\hat{P}}_{p,k|k-1}$ at time k, the state update cubature points $\chi_{p,k|k-1}^{i}$ is calculated as:

(22) $$S_{p,k|k-1}=\sqrt{{\hat{P}}_{p,k|k-1}}$$

(23) $$\chi_{p,k|k-1}^{i}={\hat{x}}_{p,k|k-1}+S_{p,k|k-1}\xi_{i}$$

$\chi_{p,k|k-1}^{i}$ is propagated through the observation equation,

(24) $${\hat{Z}}_{p,k|k-1}^{i}=h(\chi_{p,k|k-1}^{i}),i=1,2,\ldots ,2n,p=1,2,\ldots ,N$$

Further, the observation prediction ${\hat{z}}_{p,k|k-1}$ and the observation prediction error variance $P_{p,k|k-1}^{zz}$ are, respectively, obtained by

(25) $${\hat{z}}_{p,k|k-1}=\frac{1}{2n}\sum_{i=1}^{2n}{\hat{Z}}_{p,k|k-1}^{i}$$

(26) $$P_{p,k|k-1}^{zz}=\frac{1}{2n}\sum_{i=1}^{2n}({\hat{Z}}_{p,k|k-1}^{i}-{\hat{z}}_{p,k|k-1}){\left({\hat{Z}}_{p,k|k-1}^{i}-{\hat{z}}_{p,k|k-1}\right)}^{T}+R_{p,k}$$

Then, according to the probabilistic data association, the verification area of node p can be constructed by [29]:

(27) $$\left\{z_{p,k}:{\left(z_{p,k}-{\hat{z}}_{p,k|k-1}\right)}^{T}{\left(P_{p,k|k-1}^{zz}\right)}^{-1}(z_{p,k}-{\hat{z}}_{p,k|k-1})\leq \gamma \right\}$$

where γ is the gate threshold. Suppose $m_{p,k}(m_{p,k}\geq 0)$ observations fall into the validated region (27) at time k. Define validate observations ${\overset{⌣}{z}}_{p,k}$, i.e.,

(28) $${\overset{⌣}{z}}_{p,k}=z_{p,k}^{\left(j\right)},j=1,2,\ldots ,m_{p,k}$$

Actually, only one of the above observations is related to the real source; the others are due to noise or reverberation, or none of them are related to the real source. Correspondingly, for $m_{p,k}$ validated observations, there maybe be $m_{p,k}+1$ possible hypothesis, i.e.,

(29) $$\left\{ \begin{array}{l} H_{p,0}=\left\{\mathrm{All}\;\mathrm{observations}\;\mathrm{are}\;\mathrm{independent}\;\mathrm{of}\;\mathrm{real}\;\mathrm{sound}\;\mathrm{sources}\;\right\},j=0\\ H_{p,j}=\left\{z_{p,k}^{\left(j\right)}\;\mathrm{is}\;\mathrm{associated}\;\mathrm{with}\;\mathrm{the}\;\mathrm{true}\;\mathrm{source}\right\},j=1,2,\ldots ,m_{p,k} \end{array}\right.$$

According to Equation (29), the equation for calculating ${\hat{x}}_{p,k|k}^{\left(j\right)},j=0,1,\ldots ,m_{p,k}$ is as follows:

(30) $${\hat{x}}_{p,k|k}=\sum_{j=0}^{m_{p,k}}E\{x_{p,k}|H_{p,j},z_{p,1:k}\}p(H_{p,j}|z_{p,1:k})=\sum_{j=0}^{m_{p,k}}\beta_{p,k}^{\left(j\right)}{\hat{x}}_{p,k|k}^{\left(j\right)}$$

where $\beta_{p,k}^{\left(j\right)}≜p(H_{p,j}|z_{p,1:k})$ is the prior probability of event $H_{p,j}$, $0\leq \beta_{p,k}^{\left(j\right)}\leq 1$, and $\sum_{j=0}^{m_{p,k}}\beta_{p,k}^{\left(j\right)}=1$,${\hat{x}}_{p,k|k}^{\left(j\right)}≜E\left\{x_{p,k}|H_{p,j},z_{p,1:k}\right\}$ is the updated estimate conditioned on the event $H_{p,j},j=0,1,\ldots ,m_{p,k}$, and

(31) $${\hat{x}}_{p,k|k}^{\left(0\right)}={\hat{x}}_{p,k|k-1}$$

(32) $${\hat{x}}_{p,k|k}^{\left(j\right)}={\hat{x}}_{p,k|k-1}+K_{p,k}v_{p,k}^{\left(j\right)},j=1,2,\ldots ,m_{p,k}$$

where $v_{p,k}^{\left(j\right)}=z_{p,k}^{\left(j\right)}-{\hat{z}}_{p,k|k-1}$ is the innovation related to the observation $z_{p,k}^{\left(j\right)}$, $K_{p,k}$ is the Kalman gain of node p, and

(33) $$P_{p,k|k-1}^{xz}=\frac{1}{2n}\sum_{i=1}^{2n}(\chi_{p,k|k-1}^{i}-{\hat{x}}_{p,k|k-1}){\left({\hat{Z}}_{p,k|k-1}^{i}-{\hat{z}}_{p,k|k-1}\right)}^{T}$$

(34) $$K_{p,k}=P_{p,k|k-1}^{xz}{\left(P_{p,k|k-1}^{zz}\right)}^{-1}$$

where $P_{p,k|k-1}^{xz}$ is the cross covariance between the state and observation $z_{p,k}$ of node p.

Given the innovation $v_{p,k}^{\left(j\right)}$ and its covariance $P_{p,k|k-1}^{zz}$, the probability $\beta_{p,k}^{\left(j\right)}$ is generally computed as [30]

(35) $$\begin{array}{l} \beta_{p,k}^{\left(j\right)}=\{\begin{array}{c} \frac{e_{p,j}}{b_{p}+\sum_{i=1}^{m_{p,k}}e_{p,i}},j=1,2,\ldots ,m_{p,k}\\ \frac{b_{p}}{b_{p}+\sum_{i=1}^{m_{p,k}}e_{p,i}},j=0 \end{array}\\ e_{p,j}=e^{-\frac{1}{2}{\left(v_{p,k}^{\left(j\right)}\right)}^{T}{\left(P_{p,k|k-1}^{zz}\right)}^{-1}v_{p,k}^{\left(j\right)}}\\ b_{p}=\lambda |2\pi P_{p,k|k-1}^{zz}{|}^{\frac{1}{2}}\frac{1-P_{p,D}P_{G}}{P_{p,D}} \end{array}$$

where λ is the spatial probability, $P_{p,D}$ is the probability that the acoustic source is detected by sensor p, and $P_{G}$ is the gate probability.

Finally, the state estimate value ${\hat{x}}_{p,k|k}$ and error covariance ${\hat{P}}_{p,k|k}$ can be obtained by

(36) $${\hat{x}}_{p,k|k}={\hat{x}}_{p,k|k-1}+K_{p,k}v_{p,k}$$

(37) $${\hat{P}}_{p,k|k}=\beta_{p,k}^{\left(0\right)}{\hat{P}}_{p,k|k-1}+(1-\beta_{p,k}^{\left(0\right)}){\dot{P}}_{p,k|k}+{\ddot{P}}_{p,k|k}$$

where $v_{p,k}=\sum_{j=1}^{m_{p,k}}\beta_{p,k}^{\left(j\right)}v_{p,k}^{\left(j\right)}$ is the probability weighted innovation, and the covariances ${\dot{P}}_{p,k|k}$ and ${\ddot{P}}_{p,k|k}$ are respectively given by [29,30]

(38) $${\dot{P}}_{p,k|k}={\hat{p}}_{p,k|k-1}-K_{p,k}P_{p,k|k-1}^{zz}K_{p,k}^{T}$$

(39) $${\ddot{P}}_{p,k|k}=K_{p,k}\{\sum_{j=1}^{m_{p,k}}\beta_{p,k}^{\left(j\right)}(v_{p,k}^{\left(j\right)}-v_{p,k}){\left(v_{p,k}^{\left(j\right)}-v_{p,k}\right)}^{T}\}K_{p,k}^{T}$$

To summarize, the pseudo-code of the PDA-CKF method of using the observations from a single node is depicted in Algorithm 1.
**
Algorithm 1:
**
PDA-CKF Algorithm

Initialization:
${\hat{x}}_{p,0|0}={\bar{x}}_{0},{\hat{P}}_{p,0|0}=P_{0}$

Input:
${\hat{x}}_{p,k-1|k-1},{\hat{P}}_{p,k-1|k-1},z_{p,k}$

Output:
${\hat{x}}_{p,k|k},{\hat{P}}_{p,k|k}$

Iteration: for
k=1,2,…

1: Prediction step:

2: Compute the state predicted cubature points
$\chi_{p,k-1|k-1}^{i}$
at time
k−1
with (18).
3: Compute the predicted estimate ${\hat{x}}_{p,k|k-1}$ and covariance ${\hat{P}}_{p,k|k-1}$ with (20) and (21), respectively.4: Update step: 5: Compute the state update cubature points
$\chi_{p,k|k-1}^{i}$ with (23).6: Compute the predicted observations ${\hat{z}}_{p,k|k-1}$ with (25).7: Compute the innovation covariance $P_{p,k|k-1}^{zz}$ with (26).8: Select the validated observations ${\overset{⌣}{z}}_{p,k}$ according to (28).9: Compute the cross-covariance $P_{p,k|k-1}^{xz}$ with (33). 10: Compute the Kalman gain $K_{p,k}$ with (34).11: Compute the association probability $\beta_{p,k}^{\left(j\right)}$ with (35), $j=1,2,\ldots ,m_{k}$12: Compute the covariances ${\dot{P}}_{p,k|k}$ and ${\ddot{P}}_{p,k|k}$ with (38) and (39), respectively.
13: Compute the updated estimate ${\hat{x}}_{p,k|k}$ and covariance ${\hat{P}}_{p,k|k}$ with (36) and (37), respectively.

The PDA-CKF algorithm makes full use of the observation information of the node itself, which improves the tracking accuracy. However, this algorithm will fail when a node is damaged or the environmental noise and reverberation are severe. Therefore, this paper generalized PDA-CKF to a distributed version that can be used in distributed sensor networks. The improved method was named the probabilistic data association-based distributed cubature Kalman filter (PDA-DCKF). The specific process is shown in Section 3.2.

### 3.2. PDA-DCKF Algorithm

#### 3.2.1. PDA-DCKF

The neighborhood information of nodes are fused in PDA-DCKF to form local node networks. Then, the local state estimations and error covariances for the local node networks are calculated separately. Finally, the local results are fused to obtain the global state estimation.

On the basis of the above steps, the following is defined:

(40) $$\begin{array}{l} {\hat{Z}}_{Np,k|k-1}^{i}={\left[{\hat{Z}}_{p,k|k-1}^{i};{\hat{Z}}_{q,k|k-1}^{i}\right]}_{num(N_{p,k})\times 2n},i=1,2,\ldots ,2n,p=1,2,\ldots ,N,\\ q\in N_{p,k}=\{q\in \upsilon |(p,q)\in \varepsilon \}\cup \{p\} \end{array}$$

where q represents the neighborhood nodes adjacent to node p, υ={1,2,…,N} is the vertex set, ε⊂{(p,q)|p,q∈υ} is the edge set of the distributed acoustic sensor network, $num(N_{p,k})$ indicates the number of nodes in the neighborhood of node p. $N_{p,k}=\{q\in \upsilon |(p,q)\in \varepsilon \}\cup \{p\}$ denotes the set of neighbors of node p at time k, where a node is a neighbor of itself certainly.

Further, the resulting observations are fused into a matrix. Then, the observed prediction and prediction error variance are, respectively, given by

(41) $${\hat{z}}_{Np,k|k-1}=\frac{1}{2n}\sum_{i=1}^{2n}{\hat{Z}}_{Np,k|k-1}^{i}$$

(42) $$P_{Np,k|k-1}^{zz}=\frac{1}{2n}\sum_{i=1}^{2n}({\hat{Z}}_{Np,k|k-1}^{i}-{\hat{z}}_{Np,k|k-1}){\left({\hat{Z}}_{Np,k|k-1}^{i}-{\hat{z}}_{Np,k|k-1}\right)}^{T}+{\left[R_{P,k};R_{q,k}\right]}_{num(N_{p,k})\times num(N_{p,k})}$$

For single node p, $v_{p,k}^{\left(j\right)}=z_{p,k}^{\left(j\right)}-{\hat{z}}_{p,k|k-1}$ is the innovation vector related to observation $z_{p,k}^{\left(j\right)}$, and $K_{p,k}$ is the Kalman gain of node p. As far as multiple nodes are concerned, the information of node p and surrounding nodes q is fused to obtain

(43) $$P_{Np,k|k-1}^{xz}=\frac{1}{2n}\sum_{i=1}^{2n}(\chi_{p,k|k-1}^{i}-{\hat{x}}_{p,k|k-1}){\left({\hat{Z}}_{Np,k|k-1}^{i}-{\hat{z}}_{Np,k|k-1}\right)}^{T}$$

(44) $$K_{Np,k}=P_{Np,k|k-1}^{xz}{\left(P_{Np,k|k-1}^{zz}\right)}^{-1}$$

where $P_{Np,k|k-1}^{xz}$ is the cross covariance between the state and the observed value of node p after fusing the information of neighboring nodes, and $K_{Np,k}$ is the Kalman gain of node p at time k after the fusion.

The probability weighted innovation vector of local nodes is defined as

(45) $$v_{NP,k}={\left[v_{p,k};v_{q,k}\right]}_{num(N_{p,k})\times 1},p=1,2,\ldots ,N,q\in N_{p,k}=\{q\in \upsilon |(p,q)\in \varepsilon \}\cup \{p\}$$

The following is defined as

(46) $$\beta_{Np,k}^{\left(0\right)}=(\beta_{p,k}^{\left(0\right)}+\sum_{q=1}^{num(N_{p,k})-1}\beta_{q,k}^{\left(0\right)})/num(N_{p,k})$$

where $\{\sum_{j=1}^{m_{p,k}}\beta_{p,k}^{\left(j\right)}(v_{p,k}^{\left(j\right)}-v_{p,k}){\left(v_{p,k}^{\left(j\right)}-v_{p,k}\right)}^{T}\}$ is defined in the covariance ${\ddot{P}}_{p,k|k}$ of node p as $w_{p}$; when the information of node p and surrounding nodes is fused, the expression of $w_{p}$ is computed as

(47) $$\begin{array}{l} w_{Np}=\mathrm{diag}{\left(w_{p},w_{q}\right)}_{num(N_{p,k})\times num(N_{p,k})},p=1,2,\ldots ,N,\\ q\in N_{p,k}=\{q\in \upsilon |(p,q)\in \varepsilon \}\cup \{p\} \end{array}$$

where $w_{q}=\{\sum_{j=1}^{m_{q,k}}\beta_{q,k}^{\left(j\right)}(v_{q,k}^{\left(j\right)}-v_{q,k}){\left(v_{q,k}^{\left(j\right)}-v_{q,k}\right)}^{T}\}$.

Finally, the state estimate ${\hat{x}}_{Np,k|k}$ and the error covariance ${\hat{P}}_{Np,k|k}$ for node p are expressed as

(48) $${\hat{x}}_{Np,k|k}={\hat{x}}_{p,k|k-1}+K_{Np,k}v_{Np,k}$$

(49) $${\hat{P}}_{Np,k|k}=\beta_{Np,k}^{\left(0\right)}{\hat{P}}_{p,k|k-1}+(1-\beta_{Np,k}^{\left(0\right)}){\dot{P}}_{Np,k|k}+{\ddot{P}}_{Np,k|k}$$

(50) $${\dot{P}}_{Np,k|k}={\hat{p}}_{p,k|k-1}-K_{Np,k}P_{Np,k|k-1}^{zz}K_{Np,k}^{T}$$

(51) $${\ddot{P}}_{Np,k|k}=K_{Np,k}w_{Np}K_{Np,k}^{T}$$

#### 3.2.2. Fusion Strategy

After calculating the estimation of each local node in the distributed acoustic sensor network, these data need to be fused to obtain a global estimate. Since nodes in a sensor network have different reliabilities, the final tracking result integrates the estimations from the local nodes, which are weighted with the parameters depending on the mean square error of the estimation and the energy of the received signal.

(a) Energy

The energy of the signal received by each node in the acoustic sensor network is calculated [31], and the equation is described as:

(52) $$E_{p}=\underset{T\to \infty }{\lim}\int_{-T}^{T}|x_{p}(t){|}^{2}dt$$

where $x_{p}(t)$ represents the sound signal received by node p. In practice, analog signal x(t) is converted into digital signal x(n), and x(n) needs to be framed and windowed. Then, the framed signal is donated by x(n)⋅ω(n). In this paper, the Hamming window was selected for the window function ω(n). Further, the energy of each frame can be obtained by

(53) $$E_{p,n}=\sum_{m=-\infty }^{\infty }[x_{p}(m)\omega {\left(n-m)\right]}^{2}=\sum_{m=-\infty }^{\infty }{x_{p}}^{2}(m)h(n-m)={x_{p}}^{2}(n)*h(n)$$

where $h(n)=\omega^{2}(n)$, and $E_{p,n}$ represents the short-term energy of node p when the window function starts at the n−th point of the signal. The short-term energy can be regarded as the output of the square of the speech signal passing through a linear filter, and the unit impulse response of the linear filter is h(n).

(b) MSE

In Equation (48), the local estimate ${\hat{x}}_{Np,k|k}$ of node p(p=1,2,…,N) is calculated, and ${\hat{r}}_{p,k}=[\begin{array}{cccc} 1 & 0 & 0 & 0\\ 0 & 1 & 0 & 0 \end{array}]{\hat{x}}_{Np,k|k}$ is expressed as the estimated acoustic source position of node p at time k. The following is defined:

(54) $${\hat{r}}_{N,k}=\frac{1}{N}\sum_{p=1}^{N}{\hat{r}}_{p,k}$$

where ${\hat{r}}_{N,k}$ represents the global position estimation result weighted with the average consensus coefficients and calculates the MSE between the position obtained by each local node and ${\hat{r}}_{N,k}$, defined as

(55) $$M_{p}=||{\hat{r}}_{p,k}-{\hat{r}}_{N,k}|{|}^{2}$$

After calculating the energy $E_{p}$ and the mean square error $M_{p}$ of node p at time k, the following is defined:

(56) $$C_{p}=\frac{E_{p}}{M_{p}}$$

(57) $$\eta_{p}=\frac{C_{p}}{\sum_{p=1}^{N}C_{p}}$$

where $\eta_{p}$ represents the weight of node p during global fusion. A global consistency analysis is performed on the results obtained by each node according to $\eta_{p},p=1,2,\ldots ,N$:

(58) $${\hat{x}}_{k|k}=\sum_{p=1}^{N}\eta_{p}{\hat{x}}_{Np,k|k}$$

(59) $${\hat{P}}_{k|k}=\sum_{p=1}^{N}\eta_{p}{\hat{P}}_{Np,k|k}$$

To summarize, the PDA-DCKF is depicted in Algorithm 2.
**Algorithm 2:** PDA-DCKF Algorithm
Initialization:
${\hat{x}}_{p,0|0}={\bar{x}}_{0},{\hat{P}}_{p,0|0}=P_{0}$

Input:
${\hat{x}}_{k-1|k-1},{\hat{P}}_{k-1|k-1},z_{k}$
Output:
${\hat{x}}_{k|k},{\hat{P}}_{k|k}$

Iteration: for
k=1,2,…
For any node
p(p=1,2,…,N)
in sensor network

1: Prediction step:

2: Compute the state predicted cubature points
$\chi_{p,k-1|k-1}^{i}$
at time
k−1 with (18).
3: Compute the predicted estimate ${\hat{x}}_{p,k|k-1}$ and covariance  
${\hat{P}}_{p,k|k-1}$ with (20) and (21), respectively.4: Update step: 5: Compute the state update cubature points $\chi_{p,k|k-1}^{i}$ with (23).
6: Compute the observed values of predicted local nodes
${\hat{z}}_{Np,k|k-1}$
with (41).

7: Compute the innovation covariance of predicted local nodes
$P_{Np,k|k-1}^{zz}$
with (42).
8: Select the validated observations ${\overset{⌣}{z}}_{p,k}$ according to (28), p=1,2,…,N.9: Compute the cross-covariance of predicted local nodes $P_{Np,k|k-1}^{xz}$ with (43). 10: Compute the Kalman gain $K_{Np,k}$ with (44). 11: Compute the probability weighted innovation vector of local nodes $v_{Np,k}$ with (45) 12: Compute the association probability $\beta_{p,k}^{\left(j\right)}$ with (35), $j=1,2,\ldots ,m_{p,k}$. 13: Compute the association probability $\beta_{Np,k}^{\left(0\right)}$ with (46). 14: Compute $v1_{Np}$ with (47). 15: Compute the updated estimate ${\hat{x}}_{Np,k|k}$ and covariance ${\hat{P}}_{Np,k|k}$ of local nodes with (48) and (49), respectively. 16: Compute the weight $\eta_{p}$ of node p(p=1,2,…N) with (57). 17: Compute the updated estimate ${\hat{x}}_{k|k}$ and covariance ${\hat{P}}_{k|k}$ with (58) and (59), respectively.

The advantages of probabilistic data association and distributed acoustic sensor networks are combined in the PDA-DCKF proposed in this paper. In this method, the PDA algorithm is used to sift the observations from neighboring nodes. Then, the sifted observations are fused to update the state vectors in the CKF. This method not only makes the observation value obtained by each node more accurate, but also makes full use of the information of neighborhood nodes.

Meanwhile, a weighted fusion method based on local node-received signal energy and position estimation mean square error was proposed. This dynamic weighted consistency fusion considers the reliability of the local state of the nodes and provides a good global estimation performance.

## 4. Experiments and Results Discussion

To verify the performance of the proposed speaker tracking method, the evaluations are performed in a simulated room environment. Under the same conditions, the comparative experiments between PDA-DCKF and current methods are carried out, including centralized method (CCKF), DUKF, DCKF, iteration based DCKF [20] (DICKF) and DEKF. The results obtained by all methods are the average of 100 Monte Carlo runs.

The root mean square error (RMSE) is used here to evaluate the tracking performance. $r_{k}$ is expressed as the ground truth value of time k, and ${\hat{r}}_{N,k}$ represents the global consistency position calculated by the acoustic sensor network at this time. The RMSE is defined as [32]

(60) $$\mathrm{RMSE}=\sqrt{\frac{1}{K}\sum_{k=1}^{K}||r_{k}-{\hat{r}}_{N,k}|{|}^{2}}$$

where K denotes the number of frames. Generally, the smaller the RMSE, the better the tracking result.

### 4.1. Simulation Setups

**Figure 2:**
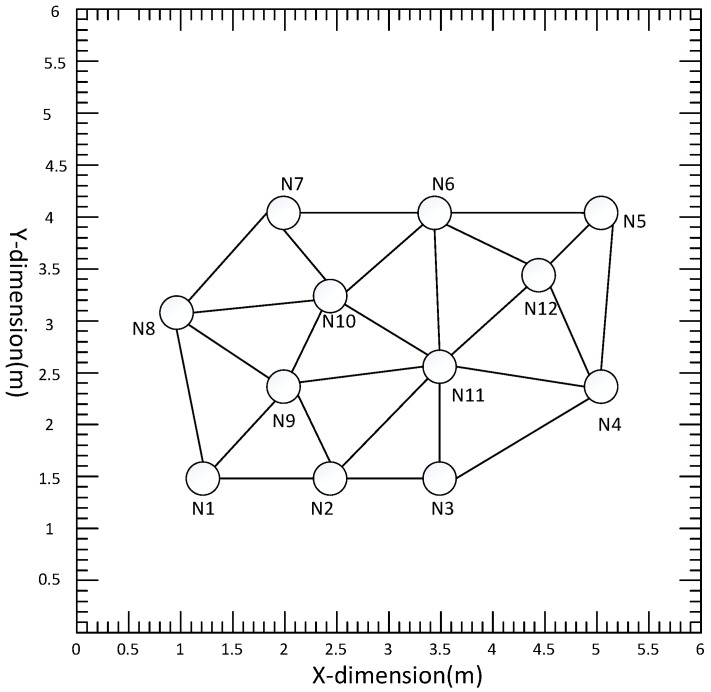
Diagram of a distributed acoustic sensor network with 12 nodes; circles represent nodes in the network. A pair of microphones was placed on each node, and the lines between the nodes indicate that the nodes can communicate with each other.

**Figure 3:**
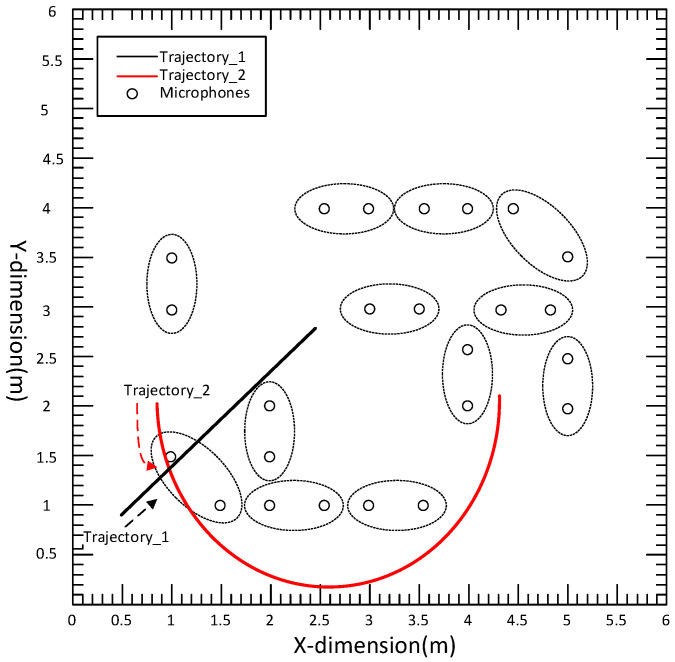
Microphone deployments and acoustic source trajectories: the black line denotes trajectory 1, the black dashed arrow denotes the motion direction of trajectory 1, the red semicircle denotes trajectory 2, the red dashed arrow denotes the motion direction of trajectory 2.

**Figure 4:**
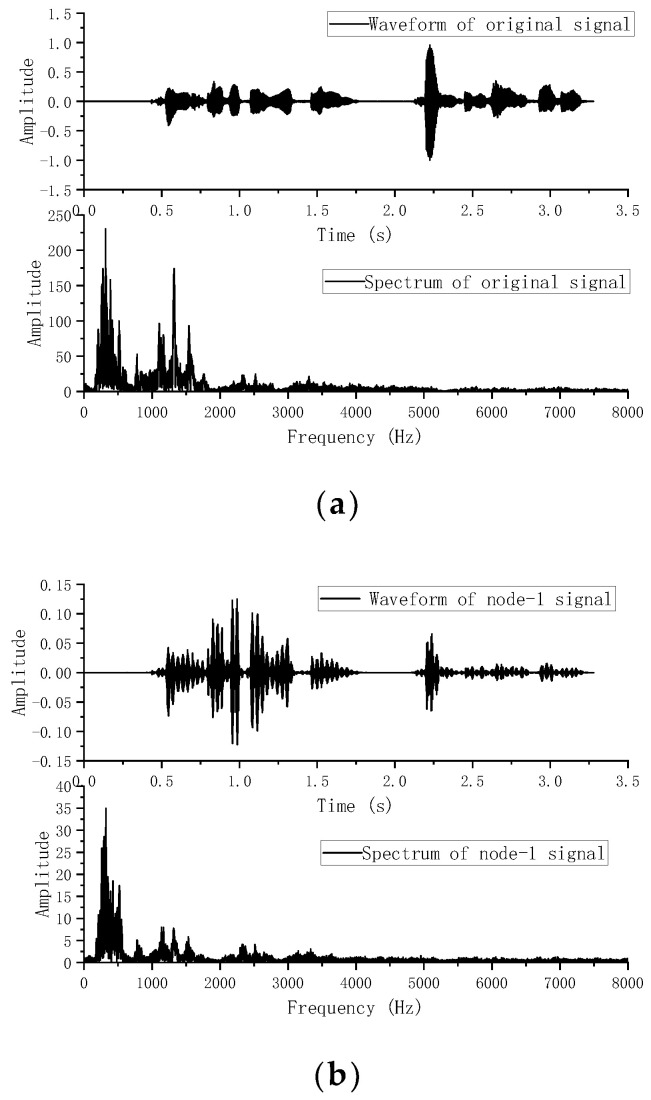
(**a**) The waveform and spectrum of the original speech signal, and (**b**) the waveform and spectrum of node 1 speech signal.

The simulation environment was a typical room of size 6 m × 6 m × 3 m, with an acoustic sensor network of 12 nodes (N=12). Each node contained a pair of microphones 0.5 m apart. The communication diagram of the distributed acoustic sensor network is shown in Figure 2, where the communication radius is 2.5 m, and each circle represents a node. The simulated trajectory 1 was a line from (0.5,0.8) to (2.5, 2.8), and trajectory 2 was an arc from (1, 2) to (4.86, 2.1), as shown in Figure 3. In different experiments, the speech sampled at the frequency of $F_{s}=16\mathrm{KHZ}$ was used as the acoustic source signal; the speech was a female recording, and the waveform and spectrum of the signal are shown in Figure 4a. The sound speed was c=342 m/s. The microphone signals were simulated with the Image method [33]. Specifically, different RIRS are generated by virtual sound source method to reflect different reverberation time. These RIRSs were convolved with the speech signal and then added to the Gaussian white noise with a determined mean and covariance to produce a received microphone signal with a mixture of reverberation and noise. The different covariance of Gaussian noise determines the different value of the signal-to-noise ratio (SNR), which reflects different environmental noise conditions. The microphone signal was divided into different signal frames along the sound source track, where the frame length of speech signal was $N_{f}=512$ and each signal frame was used for state estimation. Taking node 1 as an example, Figure 4b shows the waveform and spectrum of the speech signal received by the first microphone of node 1. For the observation TDOA, a total of eight time delays were chosen according to the magnitude of the GCC peak. From these delays, further TDOA observations were selected, where the relevant parameters were set as λ=10, γ=4, $P_{G}=0.93$, and $P_{D}=0.95$. The standard deviation of TDOA measurement error was σ=50 μs. In the acoustic dynamical model, the parameters were $\beta =10\;s^{-1}$ and $\bar{\upsilon }=1\;{\mathrm{ms}}^{-1}$. In the average consistency calculation of the global state estimation and its error covariance, the Metropolis weight was used, the number of consistency iterations [34] was $N_{con}=10$, and the number of iterations in the iterative CKF was 3.

This paper conducted four experiments to evaluate the tracking performance of PDA-DCKF. In Experiment 1, trajectory 1 was used as the acoustic source trajectory. The initial prior $p(x_{0})$ of the acoustic source position was set as a Gaussian distribution with mean $x_{0}={\left[0.5,0.8,0.02,0.02\right]}^{T}$ and covariance $P_{0}=diag([0.05,0.05,0.0025,0.0025])$. In experiment 2, the sound source signal and track were the same as experiment 1. Using simple average fusion rules, the influence of fusion rules on PDA-DCKF tracking performance was discussed. Experiment 3 discussed the robustness of the algorithm. The acoustic source and trajectory were the same as the previous two experiments. In Experiment 4, trajectory 2 was used as the acoustic source track to check the tracking results of the acoustic source when the track was nonlinear.

### 4.2. Simulation Results

#### 4.2.1. Experiment 1

**Figure 5:**
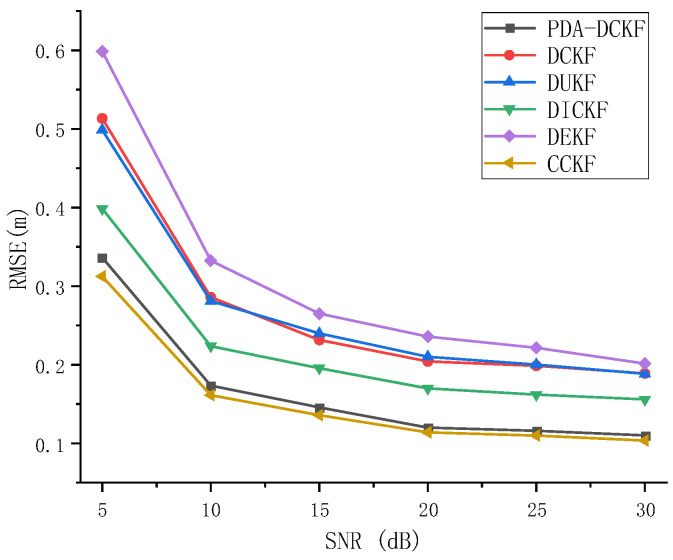
RMSE versus SNR for different tracking algorithms with *T*_60_ = 200 ms.

In this experiment, the tracking performance was evaluated under different ambient and reverberant conditions. First, the impact of environmental noise on tracking performance was investigated. Figure 5 depicts the RMSE results as a function of SNR for a reverberation time of *T*_60_ = 200 ms. In Figure 5, it is observed that the RMSE of all methods decreases with the increase of SNR, which means that the tracking accuracy increases with the increase of SNR. This is because when the SNR becomes larger, the microphone signal is less affected by ambient noise, resulting in better tracking performance. In addition, under the same SNR, PDA-DCKF performs better than traditional distributed Kalman filtering, such as extended Kalman filtering, unscented Kalman filtering, and cubature Kalman filtering. Since only one time-delayed observation of the GCC largest peak is used in traditional methods, peaks associated with real sources may be masked by spurious peaks caused by noise or reverberation, resulting in erroneous state estimates. In contrast, multiple time-difference observations of multiple largest peaks of GCC are employed in PDA-DCKF, resulting in ideal tracking performance. At the same time, compared with DICKF in this experiment, the results show that the effect of PDA-DCKF is better than that of DICKF. Because DICKF is aimed at the DCKF method, and DCKF has problems such as slow response speed and low tracking accuracy. However, the tracking performance and convergence speed of the algorithm can be improved through several local iterations in DICKF. However, still only one time-delay observation of the GCC largest peak is used in DICKF, which also causes it to be inaccurate, but as can be seen from Figure 5, as the SNR increases, the gap between DICKF and PDA-DCKF becomes smaller because the observations are more reliable when the SNR becomes larger. In addition, Figure 5 shows that PDA-DCKF is not as good as CCKF because the observation information of all nodes is used in CCKF, but PDA-DCKF achieved an effect very close to the CCKF effect, and its computational cost and the burden of the network is less than that of CCKF.

**Figure 6:**
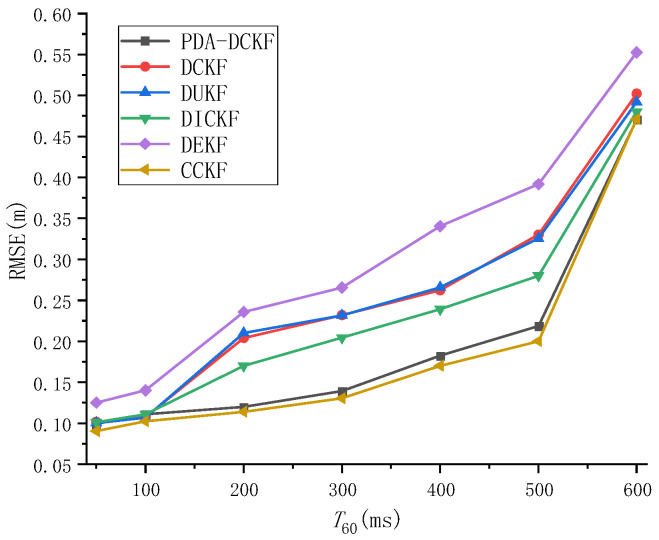
RMSE versus *T*_60_ for different tracking algorithms with SNR = 20 Db.

The effect of reverberation on tracking performance was also studied in this paper. Figure 6 depicts the RMSE results as a function of *T*_60_ with SNR = 20 dB. From the results, we can observe that the RMSEs of all the methods increased as *T*_60_ became larger, which signifies the degradation of the tracking accuracies. This may be because the microphone signal is more affected by reverberation as *T*_60_ becomes larger, the time difference observations extracted from only the largest peak or multiple largest peaks are not reliable, and the tracking performance of these methods deteriorates. In addition, it can be found from Figure 6 that the tracking performance of PDA-DCKF is better than DEKF, DUKF, DCKF, and DICKF. In fact, in traditional methods, the time-difference observations included in the scheme are only extracted from the largest peak of the GCC, while the peaks associated with the true hypocenter may be masked by false peaks caused by reverberation. In contrast, PDA-DCKF incorporates TDOA observations of multiple largest peaks of GCC into the scheme, which can alleviate the adverse effects of reverberation to a certain extent. Furthermore, the effect is not as good as CCKF showed in Figure 6, but it also achieves a very close effect.

#### 4.2.2. Experiment 2

**Figure 7:**
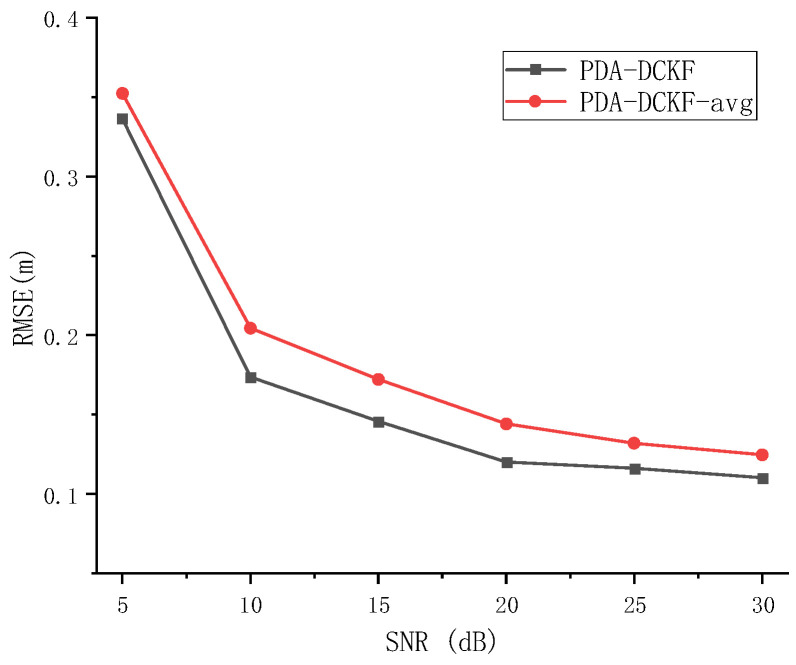
RMSE versus SNR for different fusion rules with *T*_60_ = 200 ms.

**Figure 8:**
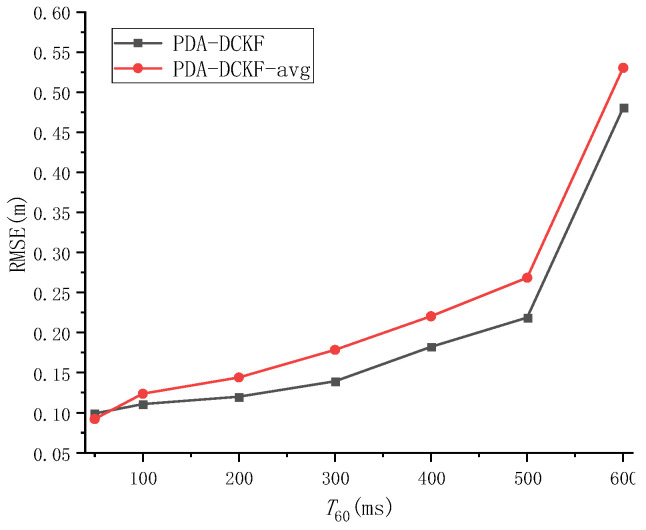
RMSE versus *T*_60_ for different fusion rules with SNR = 20 dB.

The effect of the fusion strategy proposed in this paper on the results is discussed in Experiment 2. When PDA-DCKF adopts a simple average fusion rule, it is called PDA-DCKF-avg. In this section, different SNR and different reverberations are used to test the effectiveness of the fusion strategy. The experimental results are shown in Figure 7 and Figure 8.

As depicted in Figure 7, with the increase of the SNR, the RMSEs for the PDA-DCKF methods with both these two fusion strategies decrease, but the proposed one is more effective. Figure 8 also shows that, with the increase of the reverberation time, the error also increases. In addition, only under 50 ms reverberation, the error of the average fusion strategy is smaller than that proposed in this paper, and the fusion strategy proposed in this paper was better than the average fusion effect under 100–600 ms. Comparing Figure 5, Figure 6, Figure 7 and Figure 8, it can be found that, even if the average fusion strategy is used, the PDA-DCKF in this paper is still smaller than the error obtained by the above comparison test, which further proves the effectiveness of the method in this paper.

#### 4.2.3. Experiment 3

**Figure 9:**
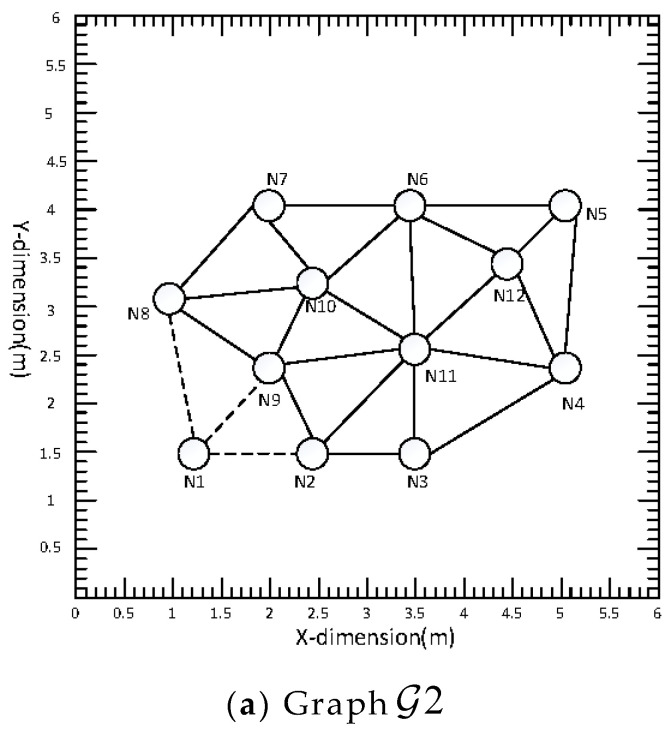

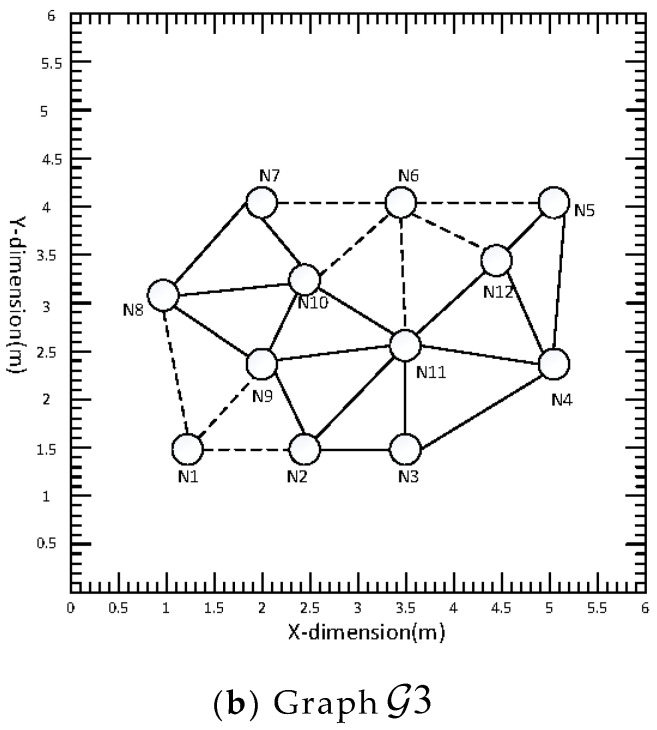
(**a**) Indicates that node 1 is broken, (**b**) indicates that node 1 and node 6 are broken. The solid line indicates that the nodes can communicate with each other, and the dotted line indicates that they cannot communicate with each other.

In practical applications, a network may be damaged by nodes, and when a node in a network is damaged, whether the network can still work normally will test the robustness of the system. In this subsection, the node damage in the distributed acoustic sensor network is simulated, and the tracking results of the acoustic source after the damage are compared with those before the damage. When node 1 in the network is damaged, it is called graph G2, as shown in Figure 9a. When node 1 and node 6 in the acoustic sensor network are damaged, it is called graph G3, as shown in Figure 9b. The experimental results are shown in Table 1 and Table 2.

It can be seen from Table 1 and Table 2 that the acoustic source can still be tracked in the case of node damage. Although the accuracy has decreased, the amplitude of the drop is not large and the acoustic source can still be tracked accurately. This can prove that the method proposed in this paper has good robustness under this network.

#### 4.2.4. Experiment 4

**Figure 10:**
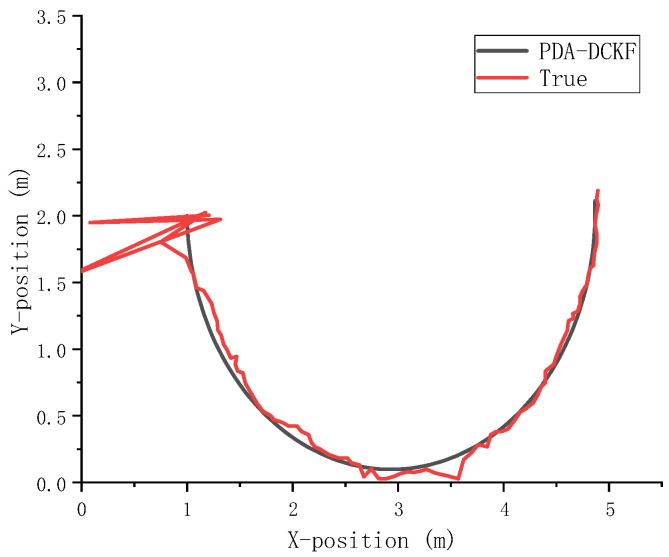
The tracking result of the semicircle trajectory when the SNR = 15 dB and *T*_60_ = 400 ms.

In order to further verify the effectiveness of the algorithm in this paper, the semicircle of trajectory 2 was used as the acoustic source trajectory, and comparative experiments were carried out under different SNR and reverberation. The experimental data are shown in Table 3 and Table 4. Figure 10 shows the tracking results with SNR = 15 dB and *T*_60_ = 400 ms.

From the above Table 3 and Table 4 and Figure 10, it can be seen that the algorithm in this paper can still accurately track the sound source in the face of such a strong nonlinear trajectory.

## 5. Conclusions

An improved PDA-DCKF method was proposed in this paper, which proved to be able to solve the problem of tracking a single mobile acoustic source with distributed acoustic sensor networks in the noise and reverberation environment. First, in order to reduce the adverse effects of noise and reverberation, the prediction value of observation is obtained by using the prediction state and the observation model of distributed nodes. Secondly, the actual observations are screened according to the predicted value. Multiple TDOA observations are extracted at each node and incorporated into the status update of CKF through PDA to generate PDA-CKF. PDA-CKF was applied to distributed acoustic sensor networks, and PDA-DCKF was further developed. In PDA-DCKF, the PDA algorithm is first used to sift the observations from neighboring nodes. Then, the sifted observations are fused to update the state vectors in the CKF. Each node runs PDA-DCKF for local state estimation and TDOA observation. Then, a new fusion strategy is proposed using energy and MSE to merge all single local estimates in a distributed manner for global state estimation. In order to apply the improved PDA-DCKF to the acoustic source tracking problem, the Langevin model was used to model the acoustic source dynamics, and a method to extract the time difference observation was proposed. Finally, a distributed acoustic source tracking framework was obtained. In order to evaluate the effectiveness of PDA-DCKF in acoustic source tracking, comparative experiments were carried out with existing methods (DCKF, DUKF, DEKF, and DICKF) under different ambient noise and reverberation conditions. The results show that the PDA-DCKF has better tracking performance than DCKF, DUKF, DEKF, and DICKF under most noise and reverberation conditions. In addition, the PDA-DCKF achieved the same tracking performance as the centralized CKF. Furthermore, it can even track the acoustic source stably in the case of node damage.

## Figures and Tables

**Table 1:** RMSE versus SNR under different graphs with *T*_60_ = 200 ms.

SNR (dB)	G1	G2	G3
5	0.3363	0.3521	0.3637
10	0.1735	0.1803	0.2057
15	0.1457	0.1511	0.1786
20	0.1201	0.1284	0.1543
25	0.1161	0.1203	0.1457
30	0.1101	0.1169	0.1376

**Table 2:** RMSE versus *T*_60_ under different graphs with SNR = 20 dB.

*T*_60_ (ms)	G1	G2	G3
50	0.0992	0.1013	0.1164
100	0.1108	0.1195	0.1351
200	0.1201	0.1284	0.1543
300	0.1393	0.1501	0.1754
400	0.1824	0.1903	0.2158
500	0.2187	0.2305	0.2439
600	0.4703	0.4897	0.5062

**Table 3:** RMSE versus SNR with *T*_60_ = 200 ms.

SNR (dB)	RMSE
5	0.3751
10	0.1869
15	0.1423
20	0.1299
25	0.1214
30	0.1167

**Table 4:** RMSE versus *T*_60_ with SNR = 20 dB.

*T*_60_ (ms)	RMSE
50	0.1174
100	0.1251
200	0.1299
300	0.1322
400	0.1635
500	0.2216
600	0.5027
